# The German Revised version of the Niigata PPPD Questionnaire (NPQ-R): Development with patient interviews and an expert Delphi consensus

**DOI:** 10.1371/journal.pone.0291002

**Published:** 2023-09-13

**Authors:** Frank Behrendt, Michaela Stark, Sarah Chételat, Stefan Schädler, Wiebke Trost, Katrin Parmar, Hans Ulrich Gerth, Leo H. Bonati, Corina Schuster-Amft

**Affiliations:** 1 Research Department, Reha Rheinfelden, Rheinfelden, Switzerland; 2 School of Health Sciences, Zurich University of Applied Sciences, Winterthur, Switzerland; 3 Translational Imaging in Neurology (ThINk) Basel, Departments of Head, Spine and Neuromedicine and Biomedical Engineering, University Hospital Basel and University of Basel, Basel, Switzerland; 4 Department of Medicine, University Hospital Münster, Münster, Germany; 5 Stroke Center and Department of Neurology, University Hospital Basel, Basel, Switzerland; 6 Department of Clinical Research, University of Basel, Basel, Switzerland; 7 Department of Sport, Exercise and Health, University of Basel, Basel, Switzerland; University of Sharjah, UNITED ARAB EMIRATES

## Abstract

**Background:**

Persistent postural-perceptual dizziness (PPPD) is a functional disorder of the nervous system and currently one of the most common types of chronic dizziness. Currently existing questionnaires do not fully assess patients’ specific symptoms of PPPD. The Japanese Niigata PPPD Questionnaire (NPQ) was recently developed following consensus-based diagnosis criteria. The aim of this study was to translate it into German, evaluate its content with the help of experts and patients and, if necessary, revise the original version to allow for a comprehensive assessment of patients’ PPPD-related symptoms.

**Methods:**

A 3-round expert Delphi survey and semi-structured patient interviews were conducted. 28 experts from Switzerland, Germany and Austria working in hospitals or outpatient centres were asked to complete a first questionnaire on various aspects of PPPD, on the translated, original NPQ and their own related experiences (Round one), a second questionnaire with statements regarding PPPD they could agree or disagree with using a 6-point Likert-scale (Round two), and a third survey to finally reach a consensus on statements to be integrated into the NPQ. In addition, eleven patients (mean age of 64.6±12.6 years; 6 females) were selected according to the criteria for the diagnosis of PPPD proposed by the Bárány Society and participated in a semi-structured interview asking for their opinion on the content of the original NPQ. All collected data were analysed using a descriptive evaluation and a qualitative content analysis based on verbatim transcripts.

**Results:**

Seven new items were added to the NPQ based on expert and patient comments and ratings. Its revised version (NPQ-R) comprises 19 items divided into five subscales using a 7-point Likert-scale with two additional subscales relating to *associated symptoms* and *symptom behaviour* in PPPD. The new maximal score is 114 points compared to 72 for the NPQ.

**Conclusion:**

The NPQ-R is the first patient-reported outcome measurement for patients with PPPD in German. It should help to provide a comprehensive assessment of the intensity of PPPD in affected patients.

## Background

Persistent postural-perceptual dizziness (PPPD) is one of the most common types of dizziness [1] and is defined as a chronic, functional disorder of the nervous system characterised by non-rotating dizziness and the perception of uncertainty or swaying [2]. Officially defined by the Bárány Society and set out in a consensus paper in 2017 [3], the term PPPD combines various previously used diagnostic terms of functional dizziness syndromes [2]. Exact data on the incidence and prevalence of PPPD are not yet available in the literature due to the lack of comprehensive epidemiological studies [4]. However, estimates can be made based on research on chronic subjective dizziness and phobic postural vertigo, which would now be classified according to the definition as PPPD [5, 6] and applies to 15 to 20% of patients with vestibular symptoms [4]. PPPD was reported as the most common diagnosis in patients up to 41 years of age [1]. Caused by a long-term maladaptation of the vestibular system to a neuro-otological, medical or psychological event, it is triggered by visual effects, in an upright posture and while moving. According to Staab et al. 2017, PPPD should be diagnosed when one or more symptoms of non-spinning vertigo, dizziness or unsteadiness are present most days for three months or longer [3, 4].

The two questionnaires that currently exist in German for assessing PPPD-related symptoms are the Dizziness Handicap Inventory (DHI) [7] and the Vertigo Symptom Scale (VSS) [8]. While the DHI was designed to gain information about everyday difficulties caused by dizziness, the VSS focuses on specific symptoms of dizziness. However, even in combination, both tests cannot satisfactorily assess the patients’ complex clinical picture of PPPD. The Japanese Niigata PPPD Questionnaire (NPQ) is capable of assessing patients’ typical PPPD-related symptoms and is suitable for the diagnosis and the determination of symptom intensity. As PPPD was defined only a few years ago, it was of clinical importance to provide such questionnaire for healthcare professionals to facilitate diagnosis. The NPQ was developed based on the diagnostic criteria of the Bárány Society [3] and validated by comparing the score differences between PPPD patients and controls. However, to ensure content validity, it is recommended to involve affected patients in the development process according to the COSMIN guidelines [9], which is, among others, an important quality criterion of questionnaires and includes the three criteria of relevance, comprehensiveness and comprehensibility. In this way, patients can contribute facts about their symptom perception and personal state of health. Furthermore, the COSMIN guidelines require a group of experts, who describe the typical signs and characteristics of the clinical PPPD picture [10]. The aim of this study was therefore to provide a revised version of the Japanese NPQ for German-speaking health professionals and their patients with PPPD for use in everyday medical practice and research. This was planned to be achieved by a) translating the original version into German and b), if necessary, by including additional items based on the expertise of patients and experts.

## Methods

Cultural differences between Japan and German-speaking countries in Europe can certainly have a significant impact on the translation and adaptation of a questionnaire in German, which refers, among other things, to communication style, social values and norms. For example, Japanese communication is said to be a bit more indirect, which influences the way questions are asked. That is why we based the translation process of the NPQ on the recommendations from Beaton et al. [11] which included two forward and two backward translations and a synthesis meeting with all four translators, three therapists, two physicians, one psychologist, and a researcher. Both doctors had several years of experience with such patients as ENT doctor or neurologist and the therapists involved had also been treating dizziness patients for many years. The other two participants had considerable experience in questionnaire development and methodology in the field of therapy research (see Table 1 for further details on the participants). Some of the translators were Japanese native speakers who offer a professional translation service but are not health professionals. The author of the original version [12] agreed to form and content of the questionnaire after the backward translation. The final version was additionally checked by a German linguist.

**Table 1:** Participants of the synthesis meeting.

Participant	Years of experience	Gender	Profession	Role in the study
1	18	f	Interpreter and translator	Backward translation
2	12	m	Interpreter and translator, Lecturer	Forward translation
3	8	f	Interpreter and translator	Backward translation
4	4	m	Interpreter and translator	Forward translation
5	25	m	Physiotherapist (PT), PPPD expert, Lecturer	Patient recruitment, Study concept
6	19	f	PT, PPPD expert	Patient recruitment
7	22	f	PT, Researcher, Lecturer	Study design, Study administrator, Synthesis meeting moderator
8	27	f	Linguist, Speech- and language therapist	Language screening/correcting
9	21	f	PT	Data collection
10	3	f	PT	Data collection
11	16	f	Physician, Oto-Rhino-Laryngology, Neck and facial surgery, PPPD expert	PPPD advisor, Delphi participant, patient recruitment
12	2	m	Physician, Lecturer	PPPD advisor, Delphi participant

We used the translated German version of the NPQ for the following study procedures. First, a Delphi survey was conducted in accordance with existing guidelines [13–16] to obtain the evaluation of the content of the current questionnaire version by a German-speaking health care professional expert panel. As a next step, qualitative interviews were conducted with PPPD-patients and finally, the findings were implemented in the German version of the NPQ, which resulted in the final revised version of the NPQ-R. The Delphi method is a widely used tool in a variety of research fields and was considered beneficial for this project. The anonymity of the experts was expected to allow open and honest expression of opinions, and the iterative nature of the method generally helps to achieve consensus and reduce the dispersion of opinions.

### 1. Delphi survey

**Fig 1:**
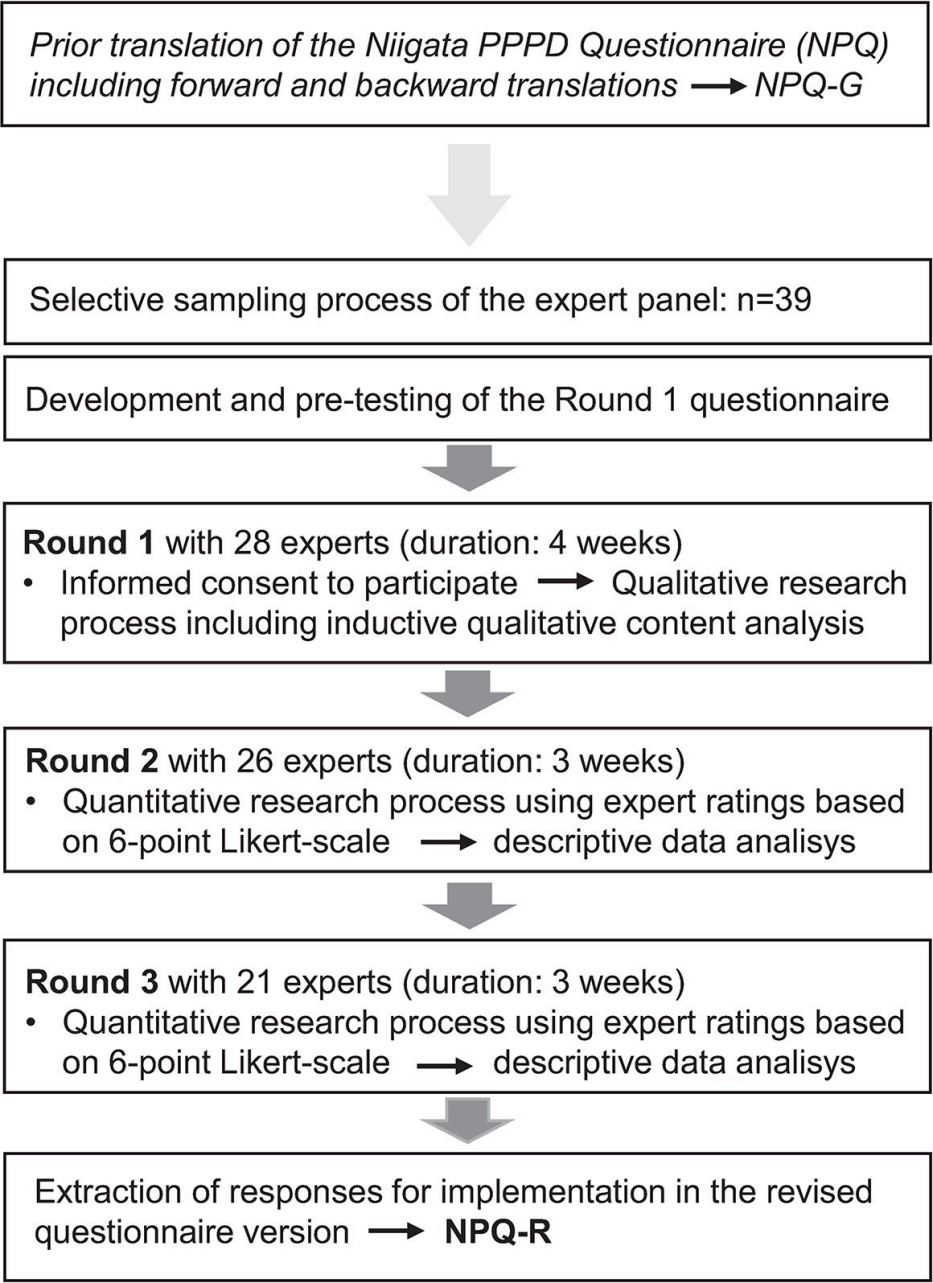
Flow chart to illustrate the stages of preparation phase and the Delphi process.

The Delphi survey is presented following the Recommendations for Conducting and REporting of DElphi Studies (CREDES) [17]. The purpose of the Delphi technique is the formation of consensus or the exploration of a field beyond existing knowledge and the current conceptual world [16]. All German-speaking experts known to us were contacted and invited to take part. The participating experts were asked on the different topics in three successive rounds using an online survey provider (see Fig 1 for the procedure). To prevent bias in the study conduct, the material that would later be provided to the experts was reviewed before by two health professionals. None of the authors had a conflict of interest.

#### Health care professional expert panel

Twenty-eight experts with extensive long-standing experience with PPPD were included. The experts had to have a minimum of five years of professional experience and continuous training on the topic of dizziness, as well as a good knowledge of German, as the survey was conducted in German. Potential experts were recruited in hospitals and outpatient centres in Switzerland, Germany, and Austria, based on a selective sampling process conducted by telephone or email. In the literature, a panel size between 10 to 50 people for a Delphi survey is recommended [16].

#### Delphi round one

For the first-round questionnaire (S1 File), open questions from three thematic fields were raised: 1. Completeness of the diagnostic criteria of the Bárány Society and the International Classification of Functioning, 2. Aspects of PPPD as reported by patients in the experts´ daily routine, and 3. Personal opinions about the content of the German version of the NPQ.

The experts were asked: a) to highlight characteristics of PPPD that, based on their experience, would be essential for implementation in a Patient Reported Outcome Measurement (PROM), b) to list characteristics of PPPD that describe a patient’s symptoms and/or are useful for documenting the progression, and c) to agree or disagree with content aspects of the NPQ or add some.

An inductive, qualitative content analysis [18] was performed to further process the experts verbatim transcribed responses using MAXQDATA (VERBI Software, Berlin, 2020) [19], a software for qualitative data analysis. Three of the authors independently coded and analysed each question and associated answers as one unit. Two of these authors (MS, SC) were students in their master’s thesis project with current training in qualitative research. The third author (CSA) had already more than 20 years of experience in qualitative and quantitative research in the field of rehabilitation.

Answers were openly coded, using the experts´ wording (in vivo codes). In the second step, all codes with the same content were integrated to subcategories. During face-to-face discussions of the categorisation deviations, a consensus was reached.

#### Delphi round two

Based on the experts’ responses, a second-round questionnaire (S2 File) was created using the defined categories and subcategories for formulating key questions and possible statements, respectively. The participating experts were asked to rate how strongly they agreed with each statement of the second-round questionnaire using a 6-point Likert-scale [20]. The questions were grouped into the four sections: symptoms/triggers, subscales, new aspects and the experts´ suggestions. Every statement that was rated with “partial agreement” or above by at least 50% of the experts was included in round three. Subsequent additions of two experts on overlaps in content were registered and considered in the questionnaire for the third-round.

#### Delphi round three

The questionnaire of the third round was nearly identical to the one of round two. Four statements with the same content were combined. For each expert, the overall ratings (percentage and quantity) and each individual rating from round two were provided for each statement. All participants were asked to re-assess every statement in the light of the group response using the Likert-scale again. Any statement that was rated as ’strongly agree’ by at least 70% of the experts or rated as ’agree’ or ’strongly agree’ by at least 90% was included in the revised version of the NPQ (NPQ-R).

### 2. Qualitative interviews

**Fig 2:**
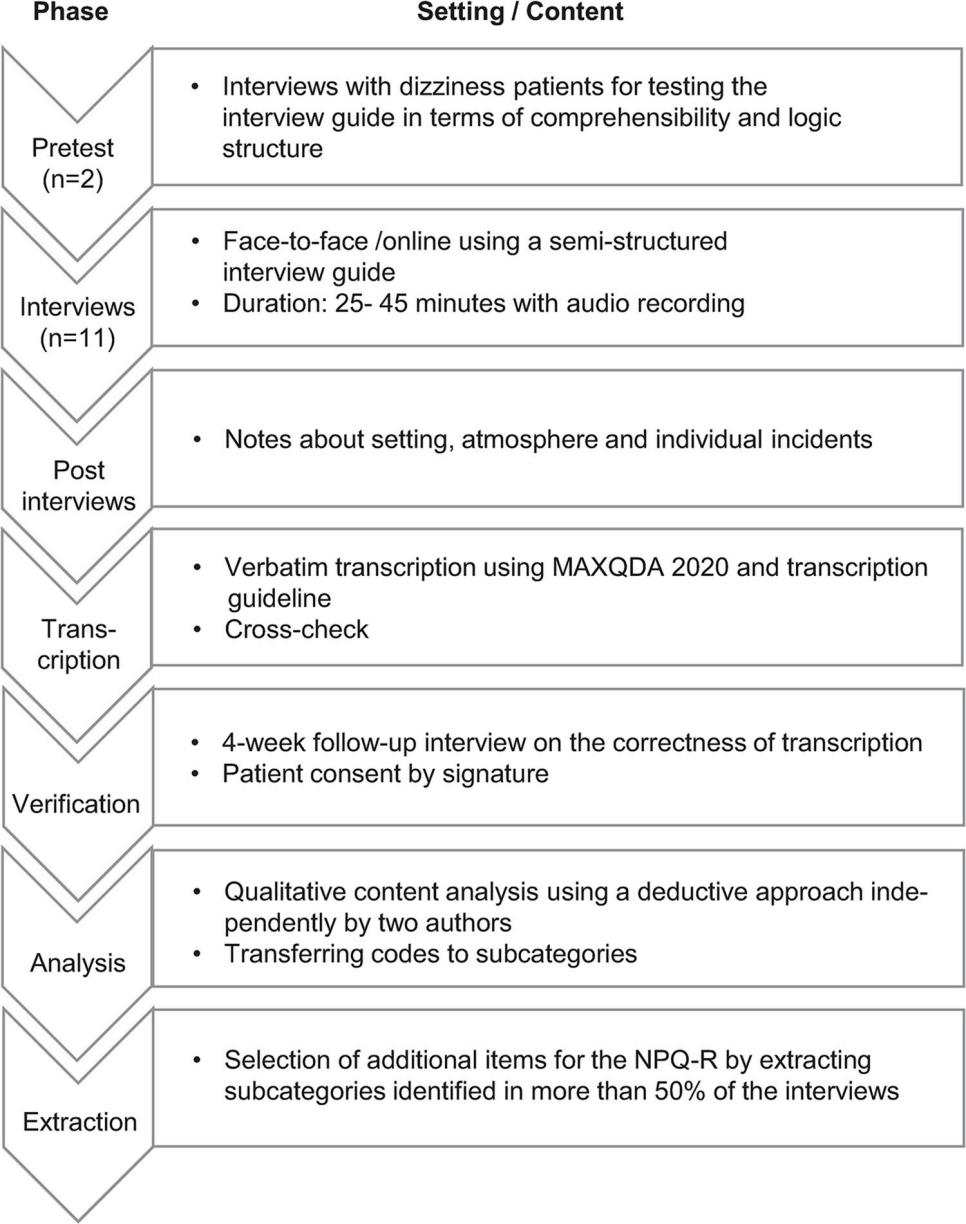
Data collection and analysis process of the qualitative interviews.

The clarification of responsiveness carried out at the Ethics Committee Northwestern and Central Switzerland confirmed that no ethics application was necessary (No 2020–01271). Patient face-to-face interviews were conducted to assess PPPD-related symptoms. The process is described below following the COnsolidated criteria for REporting Qualitative research (COREQ) [21]. Fig 2 provides an overview of all methodological steps of this sub-project with its phenomenological approach. The interview guide was tested in advance with two dizziness patients for comprehensibility and logical structure and found to be well applicable in its form.

Patients were invited for an interview if they met the diagnostic criteria for PPPD [3], were at least 18 years old and had a sufficiently good knowledge of German. Recruitment took place in various hospitals, medical practices, physiotherapy or psychological practices in the German-speaking part of Switzerland that treat patients with dizziness complaints. The interviewer who conducted the interviews was one of the authors (MS), a student who also participated in the qualitative content analysis, had 21 years of experience as a certified physiotherapist and prior experience in interviewing through her studies and work in the project. At the time of the project, she was also working in a physical therapy practice.

The sample size was determined based on literature, that suggests a minimum of seven up to 20 patients [22]. Data saturation was evaluated during the data collection process by using metrics according to the method for assessing thematic saturation [23]. The base size was set to five interviews, the run length to three interviews and the new information threshold at 0%. Written consent was obtained from all patients prior to filling in the German NPQ and the semi-structured interviews. The latter were conducted based on an interview guide (S3 File) and were audio-recorded. The audio files were verbatim transcribed and in order to give participants the possibility to review the content of the interview, the transcripts were sent within four weeks for comments and correction. Both, the interview and the coding guide used can be found in the S3 and S4 Files. For the qualitative content analysis [24], the five following interview questions on the content of the NPQ-G were selected as the units of analysis:

1. “Which questions of the PPPD-questionnaire reflect your situation very accurately or best?”
2. “Which questions can you not identify with?”
3. “What do you miss in the PPPD-questionnaire while reading it and reflecting on your own situation?”
4. “What should to be added that you can recognise yourself in it?”
5. “What would you add to the questionnaire to ensure that your situation is fully covered?”

Interviewees’ answers were paraphrased or open-coded using the patients´ wordings. For the deductive approach, we used the categories of the Delphi survey as a framework, referred to below as the deductive framework. Following a coding-guideline (S4 File), two of the authors linked the codes independently and discussed discrepancies afterwards to make the final allocations.

For data extraction, the category system and the interviews were correlated using MAXQDA (code-matrix-browser). The result was a code-matrix, which provided an overview of the number of interviews during which a statement to a category was made. Categories indicated by more than 50% of the interviews were chosen as additional items for the German NPQ-R.

### 3. Integration of new items

For the integration of the extracted statements and categories, the research team including a neuropsychologist and physiotherapists discussed content, wording and classification of the new items. The new items were phrased in a way that the numerical scale of the NPQ could be used for rating. Finally, the NPQ-R was reviewed by a linguist for the correctness of the wording.

## Results

Detailed information on the results of the Delphi rounds and the interviews can be found in the S5 and S6 Files.

### 1. Delphi survey

#### Delphi first round

Twenty-eight experts completed the first-round questionnaire (71.8% response rate, details about the experts in Table 2). Two questionnaires were excluded from the data analysis due to missing information. The first round of the survey comprised 17 questions to which a total of 390 answers were submitted (an average of 23 per question). From these answers, about 1500 codes were generated and assigned to 49 subcategories and 13 categories (S5 File). In addition, five categories were added based on individual comments on the content of the questionnaire.

**Table 2:** Information about the experts of the Delphi panel.

Details	Values	Number (n)
Panellists		28
Gender	Female Male	21 7
Age (years)	Minimum- Maximum Mean value +/- SD	36–64 50.5 +/- 8.6
Work experience (years)	Minimum- Maximum Mean value +/- SD	10–35 24.6 +/- 7.6
Profession	Physiotherapist Neurologist Physician Psychotherapist Otolaryngologist	17 5 3 2 1
Field of work (multiple answers possible)	Acute medical care Rehabilitation No comments	15 8 8
Additional qualification in the field of dizziness complaints	Yes	25
Course instructor	Yes	15
Average treated PPPD- patients within the last five years	Minimum- Maximum Median	3–500 20

#### Delphi round two

Twenty-six out of 28 experts completed the second-round questionnaire (92.9% response rate). The expert panel reached consensus in 17 categories out of 18 including 53 statements out of 54. The category including the question about previous medical or therapeutic interventions was excluded due to an agreement below 50%. All content and ratings are available as S6 File.

#### Delphi round three

Twenty-one out of 26 experts completed the third-round questionnaire (80.8% response rate). One questionnaire was excluded from data analysis, as it was incomplete. The third-round survey comprised 17 categories including 51 statements (S6 File). In this final round of the survey consensus was reached for eight statements, which were therefore chosen to be included in the new NPQ-R. The panel rated the following statements for inclusion: whether dizziness is constantly present (90.5%), influence of environment when standing (90.5%), feeling unsteady when walking (90.5%), influence of environment when walking (95.3%), standing or walking in a crowd (100%), being in a supermarket/store (95.3%), influence of distraction (95.2%) and limitation in the job (95.2%).

### 2. Qualitative interviews

The interviews were conducted in different physiotherapy practices or online via video call without another person being present. Immediately after each interview, which lasted between 25 and 45 minutes, field notes were made on the setting, atmosphere and individual incidents. No one dropped out and there were no repeat interviews. Each interview was recorded using two audio recording devices upon the written consent of the patient.

Eleven semi-structured interviews were conducted with a data saturation already achieved after eight interviews. Three more interviews were conducted to ensure not to miss any new information. However, they did not yield any new findings, therefore data collection was discontinued. All patients (mean age of 64.6±12.6 years; 6 females) checked, confirmed and returned the confirmed transcripts. One person withdrew participation due to recovery after an accident. Patients were affected by PPPD from one to 27 years and the respective, current intensity of PPPD-related symptoms ranged from one to eight on a ten-point scale (0 = none to 10 = excruciating). Further information about the patients are presented in S7 File. Five categories out of eighteen met the inclusion criteria for the NPQ-R, which resulted in three new items. Details on the analysis process of the patient interviews can be found in S8 File, and on the categories with main themes for the new items and interviewees´ quotations in S9 File.

The fourth category *Symptom aggravating factors* and the fifth category *Additions to the visual subscale* contained many different statements. Therefore, no main theme could be identified, and no content was extracted.

Categories that appeared less important for patients were *Aspects to cognition*, *Limitations on participation concerning family and friends*, and *Limitations in the execution of the profession*. The deductive framework included three categories (*Information on the initial trigger of PPPD*, *Limitations on participation*: *being dependent on help/companionship*, and *Information about previous medical clarifications or therapeutic interventions*) for which no statements were collected.

### 3. Integration of the new items

For the integration of new items, the following successive steps were carried out:

a) After checking for content overlaps, the items *Influence of the environment when standing* and *Movement in the environment* were combined into the single item *Standing or walking in a crowd*. Furthermore, the item *Being in a supermarket/store* was omitted as it is already used in the original questionnaire.
b) The content was checked for logical consistency, and the item asking whether the *Dizziness is constantly present* was rejected because it is a question that is routinely asked by health professionals to assess medical history.
c) The integration of the item *Limitation in the job* was discussed. Although this phrase reached consensus in the Delphi survey, it received low approval in the patient interviews (2/11). Since patients are the target group of the questionnaire and to reduce missing data in case persons cannot answer, e.g. because they are retired, it was finally decided to modify the wording of the item to: *When I feel dizzy*, *my performance is limited (e*.*g*. *at work*, *with childcare*, *with domestic activities)*.
d) For linguistic reasons, a new question-answer structure was created and added to the NPQ-R for four of the seven new items. In the final step, the new items were classified into two new subscales.

### 4. The NPQ-R

A total of seven new items (questions No. 3, 5, 7, 9, 10, 14, 17) were integrated. Four items resulted from the Delphi survey and three from the patient interviews.

- When I feel dizzy, I have difficulty concentrating.
- When I feel dizzy, I am frightened or anxious.
- When I give myself a break or rest, I have no (dizziness-related) problems / it is unbearable.
- When I am walking, I feel insecure.
- When I am in an unsettled environment (e.g. in a crowd, in traffic), I have no (dizziness-related) problems / it is unbearable.
- When I distract myself with an activity or turn my thoughts to something else, I have no (dizziness-related) problems / it is unbearable.
- When I feel dizzy, my performance is limited (e.g. at work, childcare, domestic activities).

The final version of the NPQ-R includes 19 items divided into five subscales (Table 3). Similar to the NPQ, patients rate on a 7-point Likert-scale. The maximal score for the NPQ-R is 114 points compared to 72 points for the NPQ.

**Table 3:** The final composition of the NPQ-R.

Number of the newly integrated items: 7 3 from patient interviews 4 from the Delphi survey	
Items total: 19	
Number of subscales: 5	
Names of existing subscales	Number of related items
*Upright posture/Standing*	4
*While moving*	4
*Visual*	4
Names of new subscales	
*Associated symptoms*	4
*Symptom behaviour*	3

## Discussion

In this project, we translated the Japanese NPQ PPPD questionnaire into German. Furthermore, we revised through a 3-stage Delphi survey and semi-structured patient interviews. The aim of this study was to provide a revised version for German-speaking health professionals and their patients with PPPD for use in everyday medical practice and research.

The survey and the interviews revealed that it was necessary to expand the existing NPQ. The data collected from the experts and patients made clear that further PPPD-related aspects needed to be considered in the questionnaire. Thus, two new subscales regarding *Symptom behaviour* and *Associated symptoms* were added, which resulted in a revised questionnaire version now comprising 19 questions. In addition to the DHI [25] and VSS [26] that evaluate dizziness-related complaints due to vestibular disorders in general, the NPQ-R provides a comprehensive PROM for patients with PPPD-related symptoms. As a result, based on Terwee et al. [22], PROM response rates may increase and responses might be more unbiased, as patients feel that their problems are extensively evaluated by the help of new version. Furthermore, because the NPQ-R indicates the intensity of PPPD, the total score and its interpretation can be used to assess and document therapy progress of fluctuations and might help to unify communication between healthcare professionals.

The three criteria of relevance, comprehensibility and comprehensiveness are important considerations when evaluating the quality of the NPQ-R. Relevance refers to how closely the content of the questionnaire relates to the intended needs of the patients. As mentioned, it is very important to be able to provide PPPD-patients with a tool that can comprehensively capture their symptoms. This is essential for the best possible medical care and successful treatment. In this respect, the expansion of the NPQ has certainly increased the relevance and importance of the questionnaire.

Comprehensibility refers to the clarity and ease of understanding that the patients have of the information being asked. In the course of the study, the patients were presented with the translated questionnaire, which had not yet been expanded at that time, in order to comment on it. At no time were there any difficulties in understanding the questionnaire, which means that it can be assumed that the extended version is also easy to understand.

With regard to comprehensiveness, it should be mentioned that with the chosen methodology we were able to obtain the expertise and experience of a sufficient number of experts and patients, and could achieve data saturation. Thus, we assume that the NPG-R covers all aspects of the PPPD syndrome.

### Strengths and limitations

An important innovation in our study was the inclusion of the patient perspective, which had not been considered before. Furthermore, the results of the study are strengthened by the number of included experts in the Delphi survey. Based on recommendations of a minimum number of 20 participants, the current study with 21 experts was sufficiently extensive, as data saturation could be achieved. Another strength of our Delphi survey certainly is the high consensus rate of 90%, which is 15% higher than reported in a corresponding review reporting 75% as median value for consensus [27].

Nonetheless, this project has also limitations. Patients were not consistently selected based solely on the medical diagnosis of PPPD. Some patients’ diagnoses included commonly used and established synonyms of PPPD, but not PPPD itself even if the criteria were met. The lack of an explicit diagnosis of PPPD could be due to the fact that the term PPPD is not yet sufficiently established in the wider medical community However, before study inclusion, patients were screened to fulfil the criteria of the Bárány society.

A second point of criticism could be that with a roughly equal gender distribution, most of the patients were over 60 years old. Thus, conclusions about limitations caused by PPPD in younger patients can only be drawn to a limited extent. Here, participation restrictions within the family situation or in the job would be more conceivable than among pensioners/retired people.

Further research is needed to assess the psychometric properties of the new NPQ-R, e.g., test-retest reliability and construct validity. In addition, the evaluation of the Minimal Important Difference would be helpful to allow clinicians to assess actual changes due to treatment interventions. In perspective, it would certainly be useful to develop and introduce a classification (mild, moderate and severe) of the intensity of PPPD based on the total score.

## Conclusion

The officially translated NPQ-R is the first disease-specific PROM for patients with PPPD in German, representing both patient and expert perspectives. As PPPD is one of the most common dizziness disorders according to current research, the new questionnaire version is likely to be helpful in assessing the intensity of PPPD and documenting the course of treatment.

## Supporting information

S1 File Delphi survey first-round questionnaire. (PDF)

S2 File Delphi survey second-round questionnaire. (PDF)

S3 File Qualitative interviews—semi-structured interview guide. (PDF)

S4 File Qualitative interviews—coding-guideline. (PDF)

S5 File Delphi survey round one–categories. (PDF)

S6 File Overview of detailed results of round 2 and 3 of the Delphi survey. (PDF)

S7 File Characteristics of the patients. (PDF)

S8 File Detailed scheme of the analysis process of patient interviews. (PDF)

S9 File Categories with main themes for the new items and interviewees´ quotations. (PDF)

## List of abbreviations

CREDES: Recommendations for Conducting and REporting of DElphi Studies
DHI: Dizziness Handicap Inventory
NPQ: Niigata PPPD Questionnaire
NPQ-G: Niigata PPPD Questionnaire- German
NPQ-R: Niigata PPPD Questionnaire- Revised
PPPD: Persistent Postural-Perceptual Dizziness
PROM: Patient Reported Outcome Measurement
PT: Physiotherapist
VSS: Vertigo Symptom Scale
