# Supplementary material for: The German Revised version of the Niigata PPPD Questionnaire (NPQ-R): Development with patient interviews and an expert Delphi consensus
Source: PLoS One. 2023 Sep 13;18(9):e0291002. doi: 10.1371/journal.pone.0291002 (PMC10499244; doi:10.1371/journal.pone.0291002)
Supplement: S1 File — (PDF) [file pone.0291002.s001.pdf]

Delphi survey: First- round Questionnaire

Section 1: Aspects of PPPD based on the diagnostic criteria of the Bárány Society and the criteria of the International Classification of Functioning (ICF).

Please answer the following questions from the point of view of whether information on the individual criteria in the questionnaire would be helpful for recording the patient's symptoms and/or suitable for documenting the course of the disease.

1. Type of symptoms
Which aspects of the symptoms do you think should be included in the questionnaire?

2. Temporal factors (for example: duration of history, frequency of symptoms, time span of symptoms, frequency per day)
What aspects of temporal factors do you think should be included in the questionnaire and why?

3. Reinforcing factors
These are included in the Niigata PPPD Questionnaire with four questions each: [...]
Are there any other items that you would like to see included in the subscale
a) "Upright/standing"? Please write them down in as much detail as possible.
Are there any other items that you would add to the subscale
b) "In motion"? Please write them down in as much detail as possible.
Are there any other items that you would add to the subscale
c) "Visual"? Please note these down in as much detail as possible.

4. Trigger and course of the PPPD
What information about the trigger of PPPD do you think should be included in the questionnaire?
What information about the course of PPPD do you think should be included in the questionnaire?

5. Burdens caused by the symptoms
Which stresses caused by the dizziness (e.g., physical stress, psychological stress, stress in everyday life, in the family, at work, etc.) do your patients talk about?

6. Functional disorders caused by the symptoms
(According to the International Classification of Functioning, these can be disturbances of body function/structure and/or limitations of activity and/or participation).
Which disturbances of body function do you notice in your patients during the examination?
What activity limitations due to dizziness do your patients talk about?
What limitations in participation in the social environment due to vertigo do your patients report?

Section 2: Aspects of PPPD as reported by patients

7. What statements are made by your patients about the type and extent of limitations in everyday life? In what frequency? (always, often, occasionally, rarely)

8. What statements are made by your patients about limitations in independence?
How often? (always, often, occasionally, rarely)

9. What do your patients say about when and what makes the dizziness worse? In what frequency? (always, often, occasionally, rarely)

10. What statements do your patients make that express aspects of PPPD that have not yet been considered? In what frequency? (always, often, occasionally, rarely)

Section 3: Personal opinions

11. What other aspects would you like to add to the Niigata PPPD Questionnaire? Please write them down in as much detail as possible - with reasons if you can.

12. Are there any general comments or suggestions you would like to make about the Niigata PPPD Questionnaire?

Socio-demographic data

- Gender
- Age
- Profession
- Work experience (in years)

- In which field do you work?
- Have you attended special training courses about dizziness or acquired additional qualifications?
- Please list here the further training courses and additional qualifications about dizziness, if possible, with the year.
- Do you give courses or trainings on the topic of "dizziness" yourself?
- Looking back over the last five years: How many people with PPPD have you treated per year on average?
