# Supplementary material for: The German Revised version of the Niigata PPPD Questionnaire (NPQ-R): Development with patient interviews and an expert Delphi consensus
Source: PLoS One. 2023 Sep 13;18(9):e0291002. doi: 10.1371/journal.pone.0291002 (PMC10499244; doi:10.1371/journal.pone.0291002)
Supplement: S2 File — (PDF) [file pone.0291002.s002.pdf]

Delphi survey: Second- round Questionnaire

Section 1: Symptoms/ Triggers

1. In your opinion, is information about the symptoms of dizziness in the questionnaire helpful for measuring the course of the disease? Yes/ no
If yes, which of the above would you include in the questionnaire? Please prioritise your statements using the rating scale.
1.1 The question of whether the dizziness is constant
1.2 Complaint-free intervals during the day
1.3 Frequency of attacks per day
1.4 Duration of attacks
1.5 Whether the dizziness occurs during or after movement.
1.6 What the dizziness feels like

2. In your opinion, is information about associated symptoms in the questionnaire helpful for measuring the course? Yes/ no
If yes, which of the above would you include in the questionnaire? Please prioritise your answers using the rating scale.
2.1 The question about headaches or migraines
2.2 The question about restricted movement or tension in the neck area
2.3 The question about nausea or discomfort
2.4 Asking about concentration problems or reduced performance

3. Do you think that questions about the initial trigger of PPPD in the questionnaire are helpful in measuring progression? Yes/ no
If yes, which of the above would you include in the questionnaire? Please prioritise your statements using the rating scale.
3.1 Acute trigger
3.2 Gradual development

Section 2: Subscales

Upright/standing subscale in the Niigata PPPD Questionnaire (questions=3,6,7,11):
3. When I walk at my own pace, then….
6. If I sit for a long time on a stool or chair without a back or armrest, then....
7. If I stand freely for a long time without holding on or leaning on, then....
11. If I walk with large steps and rather quickly, then...

4. Do you think there is a need for additions to the "Upright/Standing" subscale in the questionnaire? Yes/ no
If yes, which of the above would you include in the questionnaire? Please prioritise your statements using the rating scale.
4.1 Feeling insecure when standing

4.2 Standing looking down or moving head
4.3 Influence of the environment when standing

In motion" subscale in the Niigata PPPD Questionnaire (questions=1,5,9,12)
1. When I stand up quickly, turn around quickly, or when I make similar movements
5. When I travel by car, bus, train or other means of transport, I...
9. When I am moving around the house or doing light sport, then....
12. When I use escalators or a lift, then....

5. Do you think there is a need for additions to the "In motion" subscale in the questionnaire? Yes/ no
If yes, which of the above would you include in the questionnaire? Please prioritise your statements using the rating scale.
5.1 Walking on uneven, slippery or changing surfaces
5.2 Influence of gaze direction when walking
5.3 Walking, surrounded by people
5.4 Feeling insecure when walking
5.5 Fear of falling

Visual" subscale of the Niigata PPPD Questionnaire (questions=2,4,8,10)
2. When I look through shelves in the supermarket or hardware store, then....
4. When I see fast/hectic images in movies or on TV, then...
8. When I scroll through the screen content on a PC or smartphone, then...
10. When I read small print in books or the newspaper, then....

6. Do you think additions to the "visual" subscale in the questionnaire are necessary?
Yes/ no
If yes, which of the above would you include in the questionnaire? Please prioritise your statements using the rating scale.
6.1 Changed visibility or light conditions
6.2 Patterns on the floor or patterned fabrics
6.3 Movement in the environment
6.4 Rapid eye tracking
6.5 The absence of a visual cue

Section 3: New aspects

7. Are questions about symptom aggravating factors in the questionnaire helpful in measuring progression in your opinion? Yes/ no
If yes, which of the above would you include in the questionnaire? Please prioritise your statements using the rating scale.
7.1 Standing or walking in a crowd of people
7.2 Agoraphobic influences
7.3 Being in a supermarket/department store
7.4 Being in empty rooms or crossing a square

8. Do you think that questions about the known positive influence on symptoms in the questionnaire are helpful for measuring progression? Yes/ no
If yes, which of the above would you include in the questionnaire? Please prioritise your statements using the rating scale.
8.1 Distraction
8.2 Lying or sitting
8.3 Closing eyes to avoid visual stimuli
8.4 Movement

9. Do you think that questions about participation in the questionnaire are helpful in measuring progress? Yes/ no
If yes, which of the above would you include in the questionnaire? Please prioritise your statements using the rating scale.
9.1 Withdrawal from sports and leisure activities
9.2 Reduction in contacts with friends and family
9.3 Limitation in the execution of the profession
9.4 Childcare restrictions
9.5 Being dependent on help/companionship

10. Do you think that information on emotions related to the disease in the questionnaire is helpful in measuring the course of the disease? Yes/ no
If yes, which of the above would you include in the questionnaire? Please prioritise your statements using the rating scale.
10.1 Depressed mood
10.2 Frustration
10.3 Anxiety
10.4 Panic attacks
10.5 Feeling misunderstood
10.6 The question whether stress has an impact on symptoms

11. In your opinion, are aspects of the patient's cognition related to the disease in the questionnaire helpful in measuring progression? Yes/ no
If yes, which of the above would you include in the questionnaire? Please prioritise your statements using the rating scale.
11.1 The question of whether the cause of dizziness is feared to be a serious illness
11.2 The fact that "no cause" was found for the dizziness

12. Do you think that information on avoidance behaviour due to dizziness symptoms in the questionnaire is helpful in measuring the course? Yes/ no
If yes, would you specify an activity or would you leave a blank field where the patient can make individual entries? Please prioritise your entries using the rating scale.
12.1 Avoiding leaving the house alone
12.2 Avoiding .... (Patient can make individual entries)

13. In your opinion, are questions about previous medical clarifications or therapeutic interventions in the questionnaire helpful in measuring progress? Yes/ no
If yes, please prioritise your agreement using the rating scale.
13.1 (rating on the rating scale)

Section 4: Your suggestions

14 We would like to make specific comments from you available to the plenary. Please prioritise the extent to which you agree with the statements here using the rating scale.
14.1 to question 9 in the Niigata PPPD Questionnaire
("When I engage in household activities or light sports, I...")
Split into two questions and define "light sport".
14.2 to question 6 in the Niigata PPPD Questionnaire
("If I sit for a long time on a stool or a chair without back- or armrests, then...").
Rather no relevance
14.3 to question 5 in the Niigata PPPD Questionnaire
("When I travel by car, bus, train or other means of transport, then...").
Break down into "during" and "after".
14.4 to question 3 in the Niigata PPPD Questionnaire
("If I walk at my own pace, then...").
Add environment in which one walks (e.g. pedestrian zone, quiet street)
14.5 Suggestion to include quantification of symptoms/physical discomfort in the questionnaire.
