# Supplementary material for: The German Revised version of the Niigata PPPD Questionnaire (NPQ-R): Development with patient interviews and an expert Delphi consensus
Source: PLoS One. 2023 Sep 13;18(9):e0291002. doi: 10.1371/journal.pone.0291002 (PMC10499244; doi:10.1371/journal.pone.0291002)
Supplement: S3 File — (PDF) [file pone.0291002.s003.pdf]

Qualitative Interviews: semi-structured interview guide

Interview part	Text/ Interview questions	Follow up questions
A: Introduction	Good afternoon Mr. / Mrs. XY. Thank you for signing the consent form for the recording of the interview. It would be very helpful for the evaluation of the interview if we had an audio recording. Do you have any questions about this?<br>I would like to point out that you can stop the interview at any time without giving any reasons and request the deletion of the recording without any disadvantages for you. You also only need answer the questions that you would like to answer.<br>I would like to give you a brief overview. This interview is about finding out whether a newly translated questionnaire on chronic dizziness covers all PPPD-related problems or if something important is missing. We are particularly interested in whether you think the questionnaire covers all aspects related to your dizziness. Our interview will last about 30 minutes. As already stated in the consent form, everything you tell me will of course be treated confidentially.<br>Do you have any questions? O.K., then we'll get started.	
B: Introductory Questions	1. Is today the first time you are taking part in an interview for a scientific study?	What were the studies you participated in?<br>How do you feel in this interview situation?
	2. What expectations do you have when you have this conversation with me now?	What do you think the outcome should be?
C: Experiences with dizziness	The reason for today´s interview is the topic of chronic dizziness.	What about the dizziness can you influence yourself?<br>How can you influence the dizziness yourself?<br>How do you deal with your dizziness in everyday life?

and questionnaire	3. What experiences have you had with dizziness?	
	4. Have you recently gained other insights into your dizziness?	What have you learned from this time with the dizziness?<br>What insights do you take away from the therapy?
	5. Have you ever filled in a questionnaire?	For what purpose?
	6. Have you ever filled in a questionnaire in related to dizziness?	Can you remember the questions?<br>Why did you have to fill it in?<br>What was done with the result?
D: Transition and reading the NPQ- G	Now I ask you to take your time to read through this new questionnaire on dizziness. We will come back to the content of the questions later in the interview. If anything is not clear to you, please ask. I make a note of the time so that I don't lose track of the time frame.<br>-------------------------------<br>Now I would like to hear in your own words what you think of this dizziness questionnaire. The following questions are about finding out whether you think the questionnaire covers everything important about dizziness or whether aspects about dizziness are missing.	
E: Questions about the NPQ- G	7) Which questions of the PPPD-questionnaire reflect your situation very closely or most closely?	Why?<br>Which questions can you identify with most/best?<br>What happens when you take up different positions in an upright posture, e.g. standing or walking?<br>During which movements is the dizziness triggered - neither movements triggered by you, or movements triggered externally?<br>What trigger complex images or visual patterns that you encounter in your everyday life? For example, full supermarket shelves or successive flights of stairs?
	8. What questions can you not identify with?	Why not?<br>Which questions of the questionnaire do not apply to you?

	9. What do you miss when you read the questionnaire and think about your own situation?	Why?
	10. What would have to be added so that you can recognise yourself in it?	What other points are important for you to be able to recognise yourself in the questionnaire?<br>In your opinion, what information about the beginning of the dizziness is important to include in the questionnaire?<br>In your opinion, what information about the timing or duration of the dizziness is important to include in the questionnaire?
	11. What would you add to the questionnaire so that your situation is fully covered?	In your opinion, what information about the complaints related to dizziness is important to include in the questionnaire, e.g., the type or severity of the vertigo complaints?<br>Which stresses related to dizziness do you think are important to include in the questionnaire? E.g., mental stress, effects on family/ relatives/ profession, stress in the work environment?<br>What limitations, whether physical, in everyday life or in social life, do you think should be included in the questionnaire?
F: Summary of the answers	I would now like to summarise what you have said about the existing questionnaire. If I have understood something differently from what you meant, please correct me. Perhaps you can also think of something that you have not yet said and would like to add.<br>*(Following: summary of the answers of part E)*	
G: Final questions	Now I have the following final questions:<br>12. How would you rate the questionnaire in terms of recognising your own symptoms	

	on a scale of 0 to 10? Zero means very bad and 10 means very good?
	13. How understandable is the questionnaire for you?
	14. Is there anything we have not talked about that seems important to you in relation to your dizziness? If so, what?
H: Completion	We have now reached the end of our interview and I would like to thank you very much! On the one hand, because your descriptions have given me an insight into your very individual life situation, which is very helpful for my master's thesis. On the other hand, I would like to thank you for taking time.
