# Supplementary material for: The German Revised version of the Niigata PPPD Questionnaire (NPQ-R): Development with patient interviews and an expert Delphi consensus
Source: PLoS One. 2023 Sep 13;18(9):e0291002. doi: 10.1371/journal.pone.0291002 (PMC10499244; doi:10.1371/journal.pone.0291002)
Supplement: S4 File — (PDF) [file pone.0291002.s004.pdf]

**Qualitative Interviews: Coding- Guideline**

The following contents are assigned to the subcategories (*italics*):

*Information on dizziness symptoms:* How the patients perceive the dizziness, e.g., drowsiness, cotton wool in the head.

*Information on associated symptoms:* What other symptoms are present, e.g., neck tension, tinnitus. Symptoms that occur with or as a result of the dizziness, e.g., difficulty concentrating.

*Information on the initial trigger of the PPPD:* What triggered the dizziness. How it started.

*Additions to subscale:* Upright/standing
*Additions to subscale:* In motion
*Additions to subscale:* Visual
Statements made by interviewees that can be associated with the visual system, in motion and upright/standing and are not yet mentioned in the NPQ. These can be reinforcing factors or enhancing factors. Examples: turning the head while standing, it gets worse in the dark, it gets better with movement.

*Symptom aggravating factors:* Anything that increases the vertigo symptoms and cannot be categorised as visual, moving or upright/standing (additions to subscales of the NPQ).

*Positive influence on symptoms:* Positive influences on vertigo symptoms. All statements that improve vertigo symptoms and cannot be classified as visual, moving or upright/standing (complements to subscales of the NPQ).

*Limitations on participation:* sports/leisure time
*Limitations on participation:* Friends/family circle
*Limitations on participation:* Limitation in the execution of the profession
*Limitations on participation:* Being dependent on help/companionship
= no longer able to participate in public life (to be distinguished from avoidance behaviour!)

*Information on emotions related to PPPD:* Feelings caused by the dizziness, e.g., anxiety, depression, fainting.

*Aspects of the patient´s cognition related to the disease:* What the patient thinks about the dizziness, e.g., "there is a brain disease behind it" or "it will go away by itself".

*Avoidance behaviour:* Patients' statements about what they do not do (anymore) as activities in everyday life because of the dizziness, e.g., walking in a group, going to the playground alone with the children. (to be distinguished from limitations on participation!)

*Question 9:* Moving around the house/doing light sports

*Question 6:* Sitting longer on stool...
*Question 5:* during or after driving
*Question 3:* Complement environment in which one moves
*Question 3:* Walking at my own pace
Quantification of symptoms/complaints
Experts' statements: Here, specific questions from the NPQ are addressed. Assignments are made according to the numbers of the questions when there is a clear reference in the interview.
