# Supplementary material for: The German Revised version of the Niigata PPPD Questionnaire (NPQ-R): Development with patient interviews and an expert Delphi consensus
Source: PLoS One. 2023 Sep 13;18(9):e0291002. doi: 10.1371/journal.pone.0291002 (PMC10499244; doi:10.1371/journal.pone.0291002)
Supplement: S5 File — (PDF) [file pone.0291002.s005.pdf]

Delphi survey round one – categories

The categories assigned to four supertiles (deductive framework)

Categories	Supertitles
- Information on dizziness symptoms<br>- Information on associated symptoms<br>- Information on the initial trigger of PPPD	Symptoms/Triggers
- Additions to subscale: Upright/ Standing<br>- Additions to subscale: In motion<br>- Additions to subscale: Visual	Subscales
- Symptom aggravating factors<br>- Positive influence on symptoms<br>- Limitations on participation<br>- Information on emotions related to PPPD<br>- Aspects of the patient´s cognition related to the disease<br>- Avoidance behaviour<br>- Information about previous medical clarifications or therapeutic interventions	New aspects
- Question 9: Move around the house/Move around doing light sport.<br>- Question 6: Sitting longer on stool...<br>- Question 5: during or after driving<br>- Question 3: environment in which one moves complementary<br>- Quantification of symptoms/complaints	Your suggestions<br>(= experts´ suggestions to change questions of the NPQ)
