# Supplementary material for: The German Revised version of the Niigata PPPD Questionnaire (NPQ-R): Development with patient interviews and an expert Delphi consensus
Source: PLoS One. 2023 Sep 13;18(9):e0291002. doi: 10.1371/journal.pone.0291002 (PMC10499244; doi:10.1371/journal.pone.0291002)
Supplement: S6 File — (PDF) [file pone.0291002.s006.pdf]

## Overview of detailed results of round 2 and 3 of the Delphi survey

| **Supertitle in the survey questionnaire** | **Statement that needed to be assessed as to whether it was useful for measuring the progression of PPPD** | **Round 2: Results after summing the three scores to calculate the inclusion in round three in %** | **Round 3: Ratings of the two scores that are relevant for the calculation of consensus in %** | | **Final total leading to inclusion of the statements in the NPQ-R in %** |
|---|---|---|---|---|---|
| | | score: "partial agreement" + "agree" + "strongly agree" | score: "strongly agree" | score: "agree" | |
| Symptoms of dizziness | The question whether the dizziness is constant | 92 | 71,4 | 19,1 | 90,5 |
| | Complaint- free intervals during the day | 92 | 66,7 | 19,1 | |
| | Frequency of attacks per day | 91,6 | 40 | 30 | |
| | Duration of attacks | 96 | 33,3 | 38,1 | |
| | Whether the dizziness occurs during or after movement | 92 | 61,9 | 0 | |
| | What the dizziness feels like | 92 | 47,6 | 28,6 | |
| Associated Symptoms | The question about headaches or migraines | 84 | 38,1 | 23,8 | |
| | The question about restricted movement or tension in the neck area | 88 | 52,4 | 33,3 | |

| | | | | | |
|---|---|---|---|---|---|
| | The question about nausea or discomfort | 84 | 42,9 | 14,3 | |
| | Asking about concentration problems or reduced performance | 96 | 66,7 | 23,8 | |
| Initial trigger of PPPD | Acute trigger | 89,5 | 52,4 | 0 | |
| | Gradual development | 89,5 | 42,9 | 23,8 | |
| Additions to the "Upright/Standing" subscale | Feeling insecure when standing | 88 | 42,86 | 23,8 | |
| | Standing looking down or moving head | 88 | 19,1 | 23,8 | |
| | Influence of the environment when standing | 92 | 71,4 | 19,1 | 90,5 |
| Additions to the "In motion" subscale | Walking on uneven, slippery or changing surfaces | 86,9 | 47,6 | 14,3 | |
| | Influence of gaze direction when walking | 73,8 | 23,8 | 23,8 | |
| | Walking, surrounded by people* | 100 | see question* below | | |
| | Feeling insecure when walking | 91,3 | 71,4 | 19,1 | 90,5 |
| | Fear of falling | 95,6 | 66,7 | 28,6 | |
| Additions to the "visual" subscale | Changed visibility or light conditions | 87,5 | 47,6 | 28,6 | |
| | Patterns on the floor or patterned fabrics | 91,6 | 66,7 | 19,1 | |
| | Movement in the environment | 87,5 | 76,2 | 19,1 | 95,3 |
| | Rapid eye tracking | 79,2 | 38,1 | 14,3 | |

| | | | | | |
|---|---|---|---|---|---|
| | The absence of a visual cue | 87,5 | 47,6 | 19,1 | |
| Symptom aggravating factors | Standing or walking in a crowd of people*[a] | 100 | 90,5 | 9,5 | 100 |
| | Agoraphobic influences** | 86,9 | see question** below | | |
| | Being in a supermarket/department store | 91,4 | 76,2 | 19,1 | 95,3 |
| | Being in empty rooms or crossing a square**[b] | 78,3 | 61,9 | 19,1 | |
| Positive influence on symptoms | Distraction | 100 | 85,7 | 9,5 | 95,2 |
| | Lying or sitting | 83,4 | 61,9 | 4,8 | |
| | Closing eyes to avoid visual stimuli | 91,7 | 52,4 | 28,6 | |
| | Movement | 95,8 | 33,3 | 28,6 | |
| Questions about participation | Withdrawal from sports and leisure activities | 100 | 66,7 | 28,6 | |
| | Reduction in contacts with friends and family | 100 | 61,9 | 33,3 | |
| | Limitation in the execution of the profession | 95,8 | 71,4 | 23,8 | 95,2 |
| | Childcare restrictions | 79,2 | 28,6 | 28,6 | |
| | Being dependent on help/companionship | 100 | 66,7 | 19,1 | |

[a] the two statements marked with * were combined and provided for rating as stated "Standing or walking in a crowd of people"

[b] the two statements marked with ** were combined and provided for rating as stated "Being in empty rooms or crossing a square"

| | | | | | |
|---|---|---|---|---|---|
| Information on emotions related to the disease | Depressed mood | 83,4 | 61,9 | 28,6 | |
| | Frustration | 87,5 | 47,6 | 33,3 | |
| | Anxiety | 91,7 | 61,9 | 28,6 | |
| | Panic attacks | 86,3 | 42,9 | 28,6 | |
| | Feeling misunderstood | 75 | 33,3 | 23,8 | |
| | The question whether stress has an impact on symptoms | 95,8 | 61,9 | 23,8 | |
| Aspects of the patient's cognition related to the disease | The question of whether the cause of dizziness is feared to be a serious illness | 94,7 | 23,8 | 33,3 | |
| | The fact that "no cause" was found for the dizziness | 89,5 | 23,8 | 28,6 | |
| Information on avoidance behaviour due to dizziness symptoms | Avoiding leaving the house alone | 100 | 35,3 | 41,2 | |
| | Avoiding .... (Patient can make individual entries) | 87,5 | 70 | 15 | |
| Questions about previous medical clarifications or therapeutic interventions | yes | 48 (exclusion) | | | |
| Specific comments from panellists | To question 9 in the Niigata PPPD Questionnaire | | | | |
| | ("When I engage in household activities or light sports, I...") | | | | |

| | | | | | |
|---|---|---|---|---|---|
| | Split into two questions and define "light sport" | 60 | 9,5 | 33,3 | |
| | To question 6 in the Niigata PPPD Questionnaire | | | | |
| | ("If I sit for a long time on a stool or a chair without back- or armrests, then...") | | | | |
| | Rather no relevance | 80 | 19,1 | 14,3 | |
| | To question 5 in the Niigata PPPD Questionnaire | | | | |
| | ("When I travel by car, bus, train or other means of transport, then…") | | | | |
| | Break down into "during" and "after" | 66,7 | 19,1 | 38,1 | |
| | To question 3 in the Niigata PPPD Questionnaire | | | | |
| | ("If I walk at my own pace, then...") | | | | |
| | Add environment in which one walks (e.g., pedestrian zone, quiet street) | 76 | 23,8 | 42,7 | |
| | Suggestion to include quantification of symptoms/physical discomfort in the questionnaire | 60 | 28,6 | 14,3 | |
