# Supplementary material for: The German Revised version of the Niigata PPPD Questionnaire (NPQ-R): Development with patient interviews and an expert Delphi consensus
Source: PLoS One. 2023 Sep 13;18(9):e0291002. doi: 10.1371/journal.pone.0291002 (PMC10499244; doi:10.1371/journal.pone.0291002)
Supplement: S7 File — (PDF) [file pone.0291002.s007.pdf]

Characteristics of the patients

Interview-number	Gender	Age	Educational level	Professional education	Current work activity	Persisting dizziness for how many years?	Present intensity of dizziness on average (0= none to 10= excruciating)	Current therapeutic interventions
1	m	71	College	yes	retired	20 - 30	8	Physiotherapy
2	m	71	Secondary school	yes	retired	1	5	Physiotherapy
3	f	33	College	yes	yes	1	1- 3	Physiotherapy
4	f	75	Secondary school	no	retired	3	5	Physiotherapy
5	m	67	University diploma	yes	retired	1,5	2	Physiotherapy
6	f	61	College	yes	yes	3	5	none
7	f	76	Secondary school	no	retired	3	4	Physiotherapy
8	m	47	Secondary school	yes	yes	5 - 10	2- 3	Physiotherapy
9	f	72	Secondary school	no	retired	3	5	Physiotherapy
10	f	69	University diploma	yes	retired	27	3	Physiotherapy, therapist massage, drug (Sirdalut)
11	m	69	College of higher education	yes	retired	2	2	Physiotherapy
