# Supplementary material for: The German Revised version of the Niigata PPPD Questionnaire (NPQ-R): Development with patient interviews and an expert Delphi consensus
Source: PLoS One. 2023 Sep 13;18(9):e0291002. doi: 10.1371/journal.pone.0291002 (PMC10499244; doi:10.1371/journal.pone.0291002)
Supplement: S8 File — (PDF) [file pone.0291002.s008.pdf]

Detailed scheme of the analysis process of patient interviews

Categories	Subcategories	Interview numbers	Fullfill inclusion criteria for the NPQ-R (nominations)	Related codes (main themes are coloured)	New items for NPQ-R (nominations)
New aspects	Avoidance behaviour	3, 7, 9, 10	no (4/11)		
	Aspects of the patient´s cognition	2, 10	no (2/11)		
	Information on emotions related to PPPD	2, 3, 5, 7, 8, 9, 10, 11	yes (8/11)	galled due to dizziniess	about "anxiety" (5/8)
				initially afraid of dizziness but today not anymore (improved management)	
				at the beginning of dizziness feeling of fear and helplessness	
				to stand by dizziness costs quite an effort	
				psychological strain till you get a diagnose	
				initially adjusting with dizziness is difficult	
				not to know when it happens is depressing	
				dizziness initially strains till you get used to	
				unpredictability of dizziness stresses	
	Limitations on participation: being dependent on help/companionship		no (0/11)		
	Limitations on participation: limitation in the execution of the profession	1, 10	no (2/11)		
	Limitations on participation: friends/family circle	1, 2, 9	no (3/11)		
	Limitations on participation: sports/leisure time	2, 3, 5	no (3/11)		
	Positive influence on symptoms	2, 3, 4, 5, 7, 8, 9, 10, 11	yes (9/11)	activity	about "having a rest" (5/10)
				lying in a quiet environment and leave everything behind	
				waiting and relaxing until it gets better	

				settlement and good nutrition	
				relaxing stiff neck	
				let dizziness occur, to deviate onself	
				good diversion makes dizziness disappear	
				lying down	
				lying down, walking, fresh air	
				sitting down and relaxing the eyes	
	Symptom aggravating factors	1, 3, 5, 6, 7, 8, 9, 10, 11	yes (9/11)	the plenty of influences (traffic, people, impressions	no majority
				crowd of people	
				being in an impasse	
				riot is hard to resist	
				stress	
				stress and pressure of time	
				being in an impasse	
				flat breathing	
				insisting on an activity	
				insufficiant breathing	
				being in the mountains	
				stress	
				heat	
				city with people passing by	
				change of positions	
				drugs	
				blood pressure	
Subscales	Additions to subscale "visual"	3, 4, 7, 9, 10, 11	yes (6/11)	changing the view while car driving	no majority
				hectic pictures	
				darkness	
				duration at the PC	
				walking on and watching forest floor	
				reading for a long time	

				musters i.g. clothes, tablecloth, shutter	
				looking at bookshelves	
				musters	
	Additions to subscale "while moving"	4, 9, 10	no (3/11)		
	Additions to subscale "upright posture/standing"	5, 6, 9	no (3/11)		
Symptoms/ Triggers	Information on the initial trigger of PPPD		no (0/11)		
	Information on associated symptoms	1, 3, 7, 8, 9, 11	yes (6/11)	influence from cervical spine to dizziness	about "concentration" (3/6)
				lack of concentration	
				during strong dizziness concentration is limited	
				problems with concentration (doing something a longer time)	
				tensed neck and throat muscles	
				being imbalanced (e.g. while cycling)	
				getting tired from watching something	
	Information on dizziness symptoms	1, 2, 4, 5, 6	no (5/11)		
