# Supplementary material for: The German Revised version of the Niigata PPPD Questionnaire (NPQ-R): Development with patient interviews and an expert Delphi consensus
Source: PLoS One. 2023 Sep 13;18(9):e0291002. doi: 10.1371/journal.pone.0291002 (PMC10499244; doi:10.1371/journal.pone.0291002)
Supplement: S9 File — (PDF) [file pone.0291002.s009.pdf]

Categories with main themes for the new items and interviewees´ quotations

**Name of category**	**Main theme of the related codes (nomination)**	**Interviewees´ quotations**
1. Information on emotions related to PPPD	Anxiety (5/8)	“At the beginning of the dizziness, I felt fear and helplessness.” (Interview No. 9, 0:32:49.4)<br>“Initially, I was afraid of the dizziness, but now not anymore.” (Interview No. 10, 0:23:05.9)
2. Associated symptoms	Concentration problems (3/6)	“When you are dizzy, you are also unfocused.” (Interview No. 11, 0:27:19.9)
3. Positive influence on symptoms	Having a rest (5/8)	“Lying in a quiet environment and leaving everything behind.” (Interview No. 10, 0:11:14.4)<br>“Waiting and relaxing until it gets better.” (Interview No. 9, 0:20:31.4)
